# Validation of the COVID-19 Transmission Misinformation Scale and Conditional Indirect Negative Effects on Wearing a Mask in Public

**DOI:** 10.3390/ijerph182111319

**Published:** 2021-10-28

**Authors:** Stephen Bok, Daniel E. Martin, Erik Acosta, Maria Lee, James Shum

**Affiliations:** 1 Marketing Department, California State University, 25800 Carlos Bee Blvd, Hayward, CA 94542, USA; eacosta10@horizon.csueastbay.edu; 2 Management Department, California State University, 25800 Carlos Bee Blvd, Hayward, CA 94542, USA; daniel.martin@csueastbay.edu; 3 Department of Urban Planning and Public Policy, University of California, Berkeley, CA 92697, USA; maria.lee@me.com; 4 Accounting Department, Loyola Marymount University, Los Angeles, CA 90045, USA; jjshum14@gmail.com

**Keywords:** COVID-19 Transmission Misinformation Scale (CTMS), validation, reliability, interitem correlation, predictive validity, moderated mediation analysis

## Abstract

The SARS-CoV-2 (COVID-19) pandemic devastated the world economy. Global infections and deaths altered the behaviors of generations. The Internet acted as an incredible vehicle for communication but was also a source of unfounded rumors. Unfortunately, this freedom of information sharing and fear of COVID-19 fostered unfounded claims about transmission (e.g., 5G networks spread the disease). With negligible enforcement to stop the spread of rumors and government officials spouting unfounded claims, falsities became ubiquitous. Organizations, public health officials, researchers, and businesses spent limited resources addressing rumors instead of implementing policies to overcome challenges (e.g., speaking to defiant mask wearers versus safe reopening actions). The researchers defined COVID-19 transmission misinformation as false beliefs about the spread and prevention of contracting the disease. Design and validation of the 12-item COVID-19 Transmission Misinformation Scale (CTMS) provides a measure to identify transmission misinformation believers. Indirect COVID-19 transmission misinformation beliefs with a fear of COVID-19 decreased wearing a mask in public intentions. Callousness exacerbated COVID-19 transmission misinformation beliefs as a moderator.

## 1. Introduction

Preventative measures such as mask wearing and physical distancing have reduced the spread of COVID-19 and saved lives [1,2,3]. Unfortunately, politicians [4] and social media speculation [5] spread falsities that reduced cooperation during the early stages of the COVID-19 pandemic in the U.S.A. COVID-19 rumors rampantly spread online and became commonplace on social media newsfeeds [6]. For example, natural homeopathies (e.g., garlic) were propagated as a means to increase someone’s natural immunity to prevent contraction [7]. However, no scientific evidence has proven natural homeopathies such as garlic prevents COVID-19 infections. Belief in COVID-19 rumors reached across segments throughout society [8]. Similar to how COVID-19 did not selectively infect persons for a single predisposition, misinformation spread across society and was picked up across different groups of people. This abstract phenomenon illustrated that a psychological construct was warranted to capture this collective misunderstanding of COVID-19 transmission.

There was a misbelief that COVID-19 was just like the flu and people would receive care if they became ill [7]. Hospitals without the space and resources triaged the likelihood of survival before admitting severe cases [9,10]. The outbreak in New York City in 2020 overloaded hospitals and the likelihood of mortality was reviewed to develop triage procedures [11]. In some locations, cancer patients received a second level of triage because of limited resources and likelihood to develop severe complications [12]. COVID-19 was real. Transmission did not discriminate between ethnicities and ages [13]. One less patient in the hospital was one more open bed to someone who involuntarily contracted the disease. Misinformation endangered the public, especially when transmission was not visible and symptoms matched other common illnesses.

Months into the COVID-19 pandemic, online forums and communities reinforced false claims that researchers spent valuable time debunking [14]. COVID-19 transmission misinformation required U.S. public health organizations to create educational campaigns and webpages to counter this [15,16]. As COVID-19 shared similar symptoms to the common flu (e.g., cough and fever), perceived existence depended on trust and belief in health sciences [17]. Many contracted COVID-19 but did not develop symptoms, which further obfuscated societal acceptance [18,19]. It was projected that over 50% of new cases in 2020 were transmitted by asymptomatic COVID-19 carriers [20]. The abstract and invisible nature of the disease made transmission rumors likely to spread [21].

Social influencers shaped societal misconceptions about COVID-19 [7]. For example, in the U.S.A., politicians emphasized that COVID-19 originated in China and claimed COVID-19 was a foreign government conspiracy [22,23]. This distraction stoked racial tensions and the false belief that COVID-19 could only infect those with less civilized cleaning practices [8]. However, COVID-19 did not discriminate based on ethnicity or age [24]. This rhetoric was found to be associated with lower perceived COVID-19 threat [25]. COVID-19 conspiratorial beliefs associated with less cooperation to safety guidelines [26]. By not participating in proven safety measures, such as wearing a mask, many people unnecessarily contracted the disease. This study created the CTMS with predictive validity results on likelihood to wear a mask to demonstrate utility.

The existence of COVID-19 transmission falsehoods hindered the work of public health organizations to perform essential health related duties [27]. Debunking misinformation cost health care resources and time. For example, scientists debunked the unsupported claim that cold weather would kill the virus and it would go away [28]. Counter to this claim, COVID-19 cases increased with lower temperatures. The claim disregarded humans as warm body carriers more susceptible to diseases in lower temperatures (i.e., a basic premise of human infectious diseases). With more cases, the health care industry became overburdened with fewer resources (e.g., ventilators) to treat severe cases [29,30,31,32]. Medical and nursing students were granted accelerated courses to help overstrained facilities in Denmark [33]. Misinformation contributed to active (e.g., COVID-19 parties) and passive (e.g., live life as if there is no pandemic) contagion. A greater number of infections utilized limited health care resources to treat cases [34]. Increasing cooperation to participate in scientifically proven safety measures was a challenge for health officials during the pandemic. Therefore, the COVID-19 Transmission Misinformation Scale (CTMS) was created to encompass major transmission falsehoods perpetuated to study misinformation believers. With the CTMS, we analyzed the relationship of COVID-19 fear and callousness to access antecedents to wearing a mask in public despite holding false beliefs.

### 1.1. Theory of Rumor Dissemination

Rumors were theorized as the communication of subjective information not based on truth to explain occurrences within personal and cultural contexts [35]. Allport and Postman (1945) postulated that the degree of ambiguity and importance corresponded to the pervasiveness of rumors spreading. For instance, in the aftermath of the Pearl Harbor attacks, society was inundated with rumors [36]. In circumstances of uncertainty and anxiety, individuals try to make sense of events [37]. In these gullible times when logical facts are lacking, rumors appear believable [38,39]. “Moral panics” (i.e., travesties or perceived evildoers causing societal harm) generate a desire to share information to help [40,41]. For instance, fear for the safety of others during the initial outbreak of COVID-19 increased sharing of concocted stories of COVID-19 misinformation [42,43]. Latin doctors and hospitals were not kidnapping elderly patients for financial profits under the guise of COVID-19 infections [44]. Drinking more water does not stimulate stomach acids that kill the COVID-19 virus [45]. Rumors have pervaded overtime to fill a lack of factual data [46].

Rumors can be true or false. Rumors are part of public discourse that have historically aided in remembering information [47]. For example, stories of a berry sickening a traveler can save others from repeated illness. Fine (2007 p. 8) states, “rumors depend on other beliefs, texts and narratives for an assessment of their credibility”. Contemporary rumors reinforced by communities, forums, and multiple sources establish a culture and collective misunderstanding. When wielded for political gains, rumors can distract the public and cast doubt on existing institutions to mobilize citizens through fear [48]. For example, U.S. political claims of “fake news” were used to discredit mainstream media outlets [48]. Over time, this has created U.S. societies with lower trust and confidence in public institutions, which was found to be associated with greater belief in rumors [14]. Excessive information and suspicion in government has fostered an environment wherein citizens are more likely to believe COVID-19 transmission falsehoods.

Rumor dissemination has expanded from oral to nonoral communication [49]. The context, style, exterior shape, content, and method of propagation can affect society’s uptake [50]. Social networking platforms provided a vehicle for instantaneous hearsay diffusion through established trusted connections [51]. Epistemic naïve participants were more likely to share medical misinformation online [52]. Personal involvement, in a study on cancer rumors, increased intentions to trust and share misinformation among a medically knowledgeable experimental group [53]. Social media behaviors such as sharing and commenting are personal involvement activities. For example, participation in the Twitter post #filmyourhospital increased personal involvement, building faulty COVID-19 evidence that the pandemic was not real because hospitals were not visibly overflowing with patients [54,55]. COVID-19 rumors pose an ongoing risk to public health aggrandized by the abstract nature of epidemiology and rapid dissemination of misinformation online.

Sensationalized news instantaneously spreads in the social media marketplace because ideas compete for users’ attention [51]. There are financial incentives for click-bait-worthy news. There are social benefits to sharing misinformation (e.g., Twitter attention in the form of likes and comments). This is particularly harmful in the health field when misinformation interrupts lifesaving preventive care [56,57]. COVID-19 misinformation proliferated in society by unregulated internet websites and social media platforms. Social media fostered an online environment for transmission misbelief to permeate throughout society and communities to rapidly reinforce misunderstanding.

Falsehoods on COVID-19 transmission clouded uniformity in societal practices to combat the spread of the disease. For instance, if someone falsely believed that the mechanism of COVID-19 transmission is not through respiratory droplets spread through the air, they incorrectly reasoned face coverings would not prevent contraction. Fortunately, with advanced technology, scientists quickly studied COVID-19 transmission [34,58]. While COVID-19 misinformation became ubiquitous and took hold in society during the initial outbreak, new transmission claims did not [45]. COVID-19 transmission rumors remained constant as respiratory airborne spread was confirmed by researchers as a major source of transmission [59]. The ongoing COVID-19 pandemic warranted the development of the CTMS. Researchers need tools to study individuals prone to misinformation and additional methods to combat counterproductive misinformation dissemination.

### 1.2. Persistence of Rumors in Society despite Evidence-Based Facts

Despite counterevidence to disprove misinformation, rumors can persist in society [60]. Cultural acceptance, and socialization of ideas can enable rumors to last over time [61]. For example, discovered facts disseminated about HIV transmission had difficulty dispelling misunderstandings about contraction through the “casual contact” and “shelf life” of the virus [62]. Goldstein (2004) described the “rise of the educated lay person and the bloating of medical authority” (p. 171). A saturation of varying online information can produce resistance to medical professionals and public health policies. Regardless of personal beliefs or motives, rumors spread through interpersonal communication.

The medical community educates the public about disease prevention, benefits of treatments, and how treatments work. Unfortunately, the existence of alternative health websites with unproven statements [63], inaccurate disease transmission information [64], and vaccination folklores [65] poses public health problems that are not properly addressed. For instance, Kitta and Goldberg (2017) explained that the lack of evidence-based medical information was not the core reason people choose not to be vaccinated, but the false narratives that promote anti-vaccination behaviors. Accurate information is readily available, but there are individuals that choose to seek out and believe unproven statements online [66,67]. For example, Briggs and Hallin (2016, p. 45) described a news story framing the government as slow to respond and investigators failing in their oversight. The surfeit of public health news coverage casts doubts on what to believe [68]. This creates an environment with contradicting stories and skepticism opposing official public health messaging.

Hence, COVID-19 transmission misinformation belief was defined as those that believed falsities about how the disease was spread and prevented, not vetted as true by the scientific and public health community. For example, there was no scientific support that house flies transmit COVID-19. This rumor was fabricated but spread online through social media.

### 1.3. Fear of COVID-19 and Behavioral Changes

**Figure 1:**
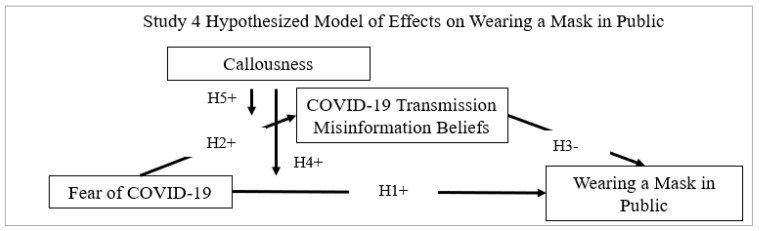
Study 4 hypothesized model of effect on wearing a mask in public.

The gravity of the COVID-19 pandemic shut down the global economy and international travel [69]. World leaders and celebrities contracted the disease [7]. News outlets reported daily infection and deaths [70]. Travesty touched the lives of millions around the world as loved ones received news of contraction or job losses. The fear of COVID-19 gripped society as health officials emphasized the risks of in-person social interactions [71]. Fear elicited cooperation to shelter-in-place and wearing masks in public for personal safety in the U.S.A. [72]. Internationally, the perceived risk of COVID-19 infection related to greater mask wearing in public among a German [73] and Vietnamese sample [74]. The fear of COVID-19 stimulated behavioral changes. Figure 1 illustrates the hypothesized relationship examined for predictive validity.

**Hypothesis** **1** **(H1).** *Higher fear of COVID-19 will relate to greater mask wearing intentions in public.*

### 1.4. COVID-19 Transmission Misinformation

Scientists identified COVID-19 transmission primarily occurring through respiratory air droplets and touching one’s face after touching a surface with the virus [20,21,75]. Misinformation was present on social media and news circuits before scientists could study the disease transmission properly in controlled settings. A problem with misinformation is difficulty unseeing misinformation [76,77]. It becomes part of someone’s thought process and judgement about an issue even when facts are presented afterwards [78]. Rumors seized hold in society regardless of whether accurate information successfully adjusted preconceived misinformation.

Thereby, public awareness of COVID-19 transmission inaccuracies remained omnipresent [45]. Despite widely available facts and debunking campaigns, misinformation believers maintained their false beliefs months later into the COVID-19 pandemic. This provided the opportunity to create the CTMS because the circulation of rumors and facts stabilized. Given COVID-19 continued to be an ongoing danger to public health, the CTMS offered a tool to identify and reach misinformation believers.

During the initial outbreak of COVID-19, more pandemic-related content was generated [17]. The public also consumed more pandemic-related media [79]. Increased social media discourse fostered an environment of fear because of unvetted information [80]. Researchers described this as an infodemic that coincided with the initial outbreak of COVID-19 [79]. Due to this uncertainty and heightened fear, it was hypothesized that fear of COVID-19 increased the consumption of misinformation.

**Hypothesis** **2** **(H2).** *Higher fear of COVID-19 will relate to greater COVID-19 transmission misinformation beliefs.*

Facial covering in public was a main recommended public health guideline to combat COVID-19 contagion [15]. Unfortunately, hearsay (such as consuming certain foods, including garlic, liquor, and pepper) permeated as means of protection. Greater belief in these rumors was expected to create a false sense of protection and reduce participation in the evidenced-based practice of mask wearing.

**Hypothesis** **3** **(H3).** *Higher COVID-19 transmission misinformation beliefs will relate to reduced mask wearing intentions in public.*

### 1.5. Callousness and Social Interactions

Callousness is a lack of emotional sensitivity and understanding of others [81,82,83]. This construct related to unfavorable romantic relationship outcomes (e.g., relationship dissatisfaction and relationship violence) [84], youth aggression [85], and psychopathic qualities with juvenile offenders [81,86]. Callousness related to lower amygdala responses to fearful expression stimuli among adolescents [87]. Disconnect from the impact of personal behaviors on others implies that callousness relates to concern for oneself first over others. Hence, greater fear of COVID-19 and callousness was postulated to increase wearing a mask in public for personal protection.

**Hypothesis** **4** **(H4).** *Callousness will moderate the relationship between fear of COVID-19 beliefs and wearing a mask in public. Higher levels of callousness and fear of COVID-19 will relate to higher mask wearing intentions in public.*

Misinformation was omnipresent during the initial COVID-19 outbreak. Accurate transmission information was second when scientific studies published findings. Callousness is a lack of emotional concern and response to others [88]. In fact, mask wearing in the U.S.A. was hotly debated as a preventative measure [89]. Health websites emphasized wearing a mask to protect others (not necessarily oneself). Such messaging muddled how someone could prevent contraction. Many COVID-19 transmission inaccuracies appeared plausible at face value (e.g., COVID-19 will go away with cold weather). Meanwhile, facts such as that COVID-19 is spread person-to-person (often from asymptomatic carriers) can be difficult to comprehend for those lacking reflective skills. Others are a source of a correcting misunderstanding. For example, someone sharing how their father contracted COVID-19 singing at a karaoke bar and dying from serious complications may not resonate with someone stiff in their beliefs about COVID-19. A callous response may be “How does this personally affect me?”. Hence, the researchers postulated that reeducating individuals with greater callousness was more difficult, and they were therefore more likely to believe debunked COVID-19 transmission misinformation.

**Hypothesis** **5** **(H5).** *Callousness will moderate the relationship between fear of COVID-19 beliefs and transmission misinformation beliefs. Greater callousness and fear of COVID-19 will relate to greater COVID-19 transmission misinformation beliefs.*

### 1.6. Overview of Studies

Psychometric theory is the foundation of instrument development for research [90,91]. The design and creation of the CTMS has several benefits to the health care industry. It lists common falsehoods for a short economical measure as a tool for identifying misinformation believers and strength of beliefs. This provides researchers with a tool for ongoing investigation of this construct.

Studies one through four followed standard practices for developing new construct measures [92,93]. Factor analysis, descriptive statistics, and consensus of misinformation beliefs were assessed across all four studies.

Studies 1 through 3 assessed multiple samples and measures for uniqueness as a new construct. These studies performed reliability, convergent validity, and discriminant validity analysis.

Study 4 conducted similar analyses as the previous studies. In addition, study 4 extended results by also collecting dependent variable data (i.e., wearing a mask in public) to evaluate predictive validity using moderated mediation analysis. Predictive validity advances utility of the measure for future research.

## 2. Method

### 2.1. Overview of Studies

To establish consistency and validity of the CTMS, the researchers conducted four studies with online participants using the survey platform Qualtrics. Four studies allowed for the collection of a wide variety of variables to analyze the convergent, discriminant, and predictivity validity otherwise too long for one study (i.e., cause fatigue and potential surveying error) [94,95].

Recruitment was through the Amazon Mechanical Turk (MTurk) system. Qualified participants viewed standard postings and registered by choice. Participants could only participate in one of the four studies. Amazon Mechanical Turk participants represented all states in the U.S.A. Participants had HIT approval rates of over 97%, which was found to relate to greater accuracy to attention checks and data quality (recommended over 95%) [96]. Surveys were completed nationally by U.S. samples during January and February of 2021 over two-week periods. A wide range of multidisciplinary MTurk studies have been conducted and recognized as suitable for generalizable sampling [97]. Psychometric development followed standard scale development processes [92,98,99]. Phase 1 constructed and refined measure items. Phase 2 analyzed the reliability, convergent validity, and discriminate validity. Phase 3 assessed the predictive validity (study 4).

All studies collected responses to the initial 28 items to collate large datasets for varying psychometric analyses. After completing consent, participants completed the full list of CTMS statements, battery of individual difference measures, and demographic questions. Study 4 additionally collected intent on wearing a mask in public for predictive validity analysis. Each of the studies collected different variables to evaluate discriminant and convergent validity with a variety of validated scales.

### 2.2. Phase 1 Initial Item Generation

The researchers generated the initial list of items for the psychometric design by reviewing the widely held COVID-19 transmission falsehoods [7,100] and reputable public health webpages debunking rumors [16,75]. Well-known falsehoods spread during the first outbreak when it was not clear how the disease was transmitted. Conspiracy-prone social media users spread speculatory COVID-19 transmission inaccuracies that lacked fact-based evidence [5]. The researchers carefully worded each statement to succinctly express one misconception [93]. A rigorous vetting process was performed to assess if the statements fit the criteria for COVID-19 transmission misinformation beliefs. Only those that were considered COVID-19 transmission misinformation related remained in the initial 28-item list.

All survey participants across the four studies rated COVID-19 transmission misinformation statements on a 5-point scale (1—definitely false, 2—probably false, 3—not sure/cannot decide, 4—probably true, 5—definitely true). This followed the same scale points and coding of the Generic Conspiracy Belief Scale, which distinguished between facts and fiction [101]. Each item was randomly presented within the section to minimize ordering bias. Schmidt et al. (2003) and Donnellan et al. (2006) studied the reliability of long forms of measures with short forms. These researchers found short forms of measures to have overall similar reliability scores for the same construct, designed to adequately measure the construct while reducing problems with lengthy surveys [94,95]. In continuous true/false scales, those that slightly believe as true and above are considered believers [101,102]. Most agreeing on statements is ideal for these new objective scales [102].

### 2.3. Participant Inclusion

Completion of the survey and attention checks determined inclusion. Of 2513 total participants over the four studies to attempt the survey, 51 did not complete the survey and 29 failed the attention checks. A total of 2433 online participants remained. For scale development studies, samples over 300 participants are “generally sufficient in most cases” [99]. An estimated 385 or more participants was needed for a confidence level of 95% with a real value within ±5% of the measured value for a generally large population. Each study exceeded the suggested and estimated sample size.

### 2.4. Inter-Item Correlations and Reduction

Across the four studies, evaluation of the 28 items for inter-item correlations were acceptable for 15 items. Exclusion of 13 items was based on low inter-item correlations (|r|s < 0.30) [103]. Items with scores lower than this threshold were removed from the initial list following guidelines for item reduction in scale development [99]. For example, despite the politically popularized misconception that “Facemasks are useless to prevent COVID-19 infection”, this item was low on the inter-item correlation threshold. Items displaying values below 0.30 were removed at this time using objective and statistical results to refine the list. Kaiser–Meyer–Olkin (KMO) results showed satisfactory sampling adequacy (see Table 1). Results of Bartlett’s test of sphericity indicated items were suitable for factor analysis.

### 2.5. Factor Loadings

Parallel analysis and evaluation of the scree plot showed items adequate as one factor. The first item explained 71.95% of the common variance. Two principal component factor loadings (direct oblimin rotated) analysis produced scores beneath 0.20 for the second factor. Analysis of items forced into two factors provided inadequate scores [90,104]. The researchers assessed the new measure appropriate as one factor.

Factor loadings are recommended to have scores above the 0.40 minimum [105,106]. More conservative factor loadings top 0.70 [107,108]. Twelve items topped 0.70 factor loading scores. Table 2 illustrates the component scores of the 12-item CTMS retained for deeper analysis.

### 2.6. COVID-19 Transmission Misinformation Consensus

The transmission rumors presented to participants were debunked by the scientific community and not proven true by reputable researchers/sources [16,75]. Responses rated 1—definitely false, 2—probably false, and 3—not sure/cannot decide were counted as believe as not true (i.e., dummy coded as 0). Responses rated 4—probably true and 5—definitely true were counted as believe as true (i.e., dummy coded as 1). This followed the dichotomous scoring method used for generic conspiracy beliefs to evaluate percentage of the sample to believe statements as false [101]. Table 3 illustrates frequencies and percentages of the twelve items believed not true. The percentages were out of 2433 total participants over the four studies. A consensus represents the level of agreement for appropriate construction of an objective measure [102]. For true/false measures, Clark and Watson (1995) stated that “virtually everyone (e.g., 95% or more) either endorses or denies” statements with Likert formatted ratings producing similar results. The CTMS items had over 80% consensus with many approaching 90%. This was overall high, given the range of news sources and variety of misinformation spread during the COVID-19 pandemic. Skewness (less than ±2) and kurtosis (less than ±7) was within acceptable ranges that did not substantially depart from normality [109,110]. The CTMS was intended as a specific true/false type scale to identify misinformation believers based on the large sample and this consensus.

### 2.7. Demographic Variables

The researchers collected demographic information regarding gender, age, education, average weekly hours of news watched, and level of religiosity. Education was assessed by the highest level of degree completed. Religiosity was measured averaging two items (“Religion/spirituality was an important part of my up bringing” and “I currently consider myself to be a member of a religious or spiritual organization”) on a 7-point scale (strongly disagree—1 to strongly agree—7).

Table 4 illustrates demographic frequencies and percentages. Of the total number of participants across the four studies (*N* = 2433), 65% were female. The average age was 41 years old (range = 19–88 years old). An associate degree or higher was earned by 70% of participants. Each week on average 3.84 h of news was watched. Participants averaged 4.25 on the 7-point religiosity scale. The participants represented the ethnically diverse U.S.A. with 1915 Caucasians, 118 Hispanics/Latinos, 208 African Americans, 26 Native Americans, 126 Asians, and 40 identifying as other.

## 3. Results

### 3.1. Phase 2 Reliability

Table 5 displays reliability, variable means, standard deviations, and correlations to the CTMS in each of the four studies. Across the four studies, high reliability was present. These results evinced the 12-item CTMS had high internal consistency. The 12-items represented a wide range of different unfounded claims on COVID-19 transmission circulated, from treatment by drinking bleach and spread from houseflies. The high reliability demonstrated that participants showed high consistency in believing or not believing the gamut of false statements. High reliability scales have demonstrated acceptable construct validity to measure what they intended [111,112,113].

### 3.2. Convergent and Discriminant Validity

Correlations across a variety of measures were examined to assess discriminant validity and uniqueness as a measure [114,115]. The researchers correlated the CTMS with the following measures: fear of COVID-19 (i.e., degree of fear to the novel coronavirus) (alpha = 0.82) [116], callousness (i.e., rigidity in ideas) (alpha = 0.85) [81,88], conscientious (i.e., following rules/norms) (alpha = 0.81) [117], conservatism (i.e., right-wing ideology) (alpha = 0.83) [118], generic conspiracy beliefs (i.e., credence to unproven conspiracies) (alpha = 0.95) [101], Positive and Negative Affect Schedule (PANAS) (i.e., state of emotions) (alpha = 0.87) [119], perceived vulnerability to disease (i.e., estimated harm of illnesses) (alpha = 0.82) [120], variety seeking (i.e., diversity in selection) (alpha = 0.81) [121], risk taking (i.e., exposure to threats/dangers) (alpha = 0.85) [83], and compassion (i.e., concern for suffering) (alpha = 0.84) [122]. Correlation is not evidence to support causation. However, it does indicate the degree of relatedness and evidences that a measure differs from another [123]. Measures significantly correlated with the 12-item CTMS. The results supported expected relationships with other measures. Across the four studies, fear of COVID-19 demonstrated a consist middling correlation with the CTMS.

Conscientious (study 1: r = −0.408, *p* < 0.001; study 2: r = −0.422, *p* < 0.001), variety seeking (study 3: r = −0.092, *p* < 0.001), and compassion (study 4: r = −0.301, *p* < 0.001) demonstrated a negative correlation with the CTMS. Meanwhile, conservatism (study 1: r = 0.164, *p* < 0.001; study 2: r = 0.139, *p* < 0.001) and generic conspiracy beliefs (study 1: r = 0.605, *p* < 0.001; study 2: r = 0.556, *p* < 0.001) positively correlated with the CTMS.

Further, callousness (study 3: r = 0.789, *p* < 0.001; study 4: r = 0.736, *p* < 0.001) demonstrated a positive correlation with the CTMS. Higher CTMS scores correlated with higher risk-taking scores (study 3: r = 0.750, *p* < 0.001; study 4: r = 0.678, *p* < 0.001) and lower personal vulnerability to disease scores (study 3: r = −0.092, *p* < 0.001; study 4: r = −0.106, *p* < 0.001). A wide range of correlated measures provided support for convergent and discriminant validity.

### 3.3. Study 4 Moderated Mediation Analysis

#### Phase 3 Measures

Independent variable. Fear of COVID-19 measures the degree of COVID-19 phobia using seven items on a five-point scale from (1—strongly disagree to 5—strongly agree) (alpha = 0.88) [116]. For example, an item states: “My heart races or palpitates when I think about getting coronavirus-19”.

Dependent variable. Wearing a mask in public around others and in enclosed spaces (e.g., grocery stores) was mandated because of knowing the transmission method of COVID-19 infection. Wearing a mask in public was a fundamental practice to decrease communal spread of the disease [124]. The intention to wear a mask in public was measured on a seven-point scale (“I wear a face covering in public” from 1—strongly disagree to 7—strongly agree).

Moderator. Callousness is the degree someone lacks empathy and sympathy for others [81,87]. It was measured using the CAT-PD short seven-item scale (e.g., “Do not care how my actions affect others”). The measure demonstrated high reliability (alpha = 0.85).

### 3.4. Predictive Validity Results

**Figure 2:**
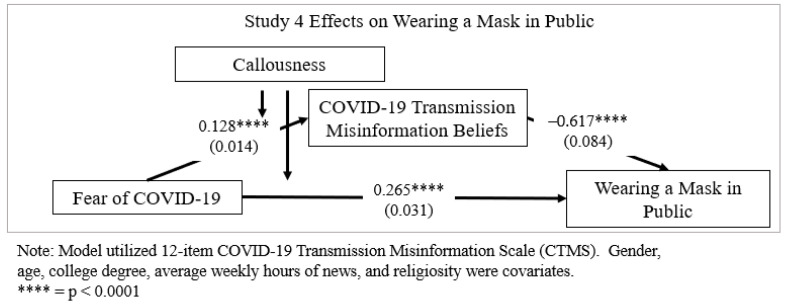
Study 4 effects on wearing a mask in public model estimates.

Moderated mediation analysis was conducted using SPSS PROCESS V3.5 software [125,126] (see Table 6 and Figure 2). The 10,000 bootstrapped sampling procedure was applied for estimations [127]. There was mean centering of variables. Statistical significance was considered at the 95% confidence interval and when zero was not in between confidence intervals. Wearing a mask in public was overall high among participants on the seven-point scale (*M* = 6.36, *SE* = 1.19).

A omnibus test of moderated mediation of callousness and COVID-19 transmission misinformation beliefs demonstrated a significant indirect effect with fear of COVID-19 on wearing a mask in public (*effect* = −0.077, *SE* = 0.014 (LLCI − 0.108 ULCI − 0.052)) [127,128]. Greater fear of COVID-19 was associated with greater masking wearing in public (*t*(592) = 8.496, *SE* = 0.031, *p* < 0.0001 (LLCI 0.203 ULCI 0.326)) (H1). Greater fear of COVID-19 was associated with greater COVID-19 transmission misinformation beliefs (*t*(593) = 8.973, *SE* = 0.014, *p* < 0.0001 (LLCI 0.010 ULCI 0.156)) (H2). Greater COVID-19 transmission misinformation belief was associated with decreased mask wearing in public (*t*(592) = −7.315, *SE* = 0.084, *p* < 0.0001 (LLCI − 0.782 ULCI − 0.451)) (H3) [129]. Someone fearful of COVID-19 expressed greater intent to wear a mask in public; however, in the presence of greater belief in COVID-19 transmission misinformation, their intent decreased. In other words, those more fearful who reviewed and believed transmission rumors displayed lower mask wearing intent. For example, someone fearful but who ate garlic the night before may be less concerned about wearing a mask for protection because they falsely believe garlic protects against transmission. Adequate fit was demonstrated based on SPSS AMOS V25 modeling following recommended thresholds (χ^2^/df = 3.998, *p* < 0.0001, RMSEA = 0.071, SRMR = 0.077, CFI = 0.943) [130,131].

The researchers included age, gender, college degree, average weekly hours of news, and religiosity as covariates in the moderated mediation model. The model with and without the covariates shared the same pattern of statistical significance and direction of relationships.

### 3.5. Moderated Mediation Results

**Figure 3:**
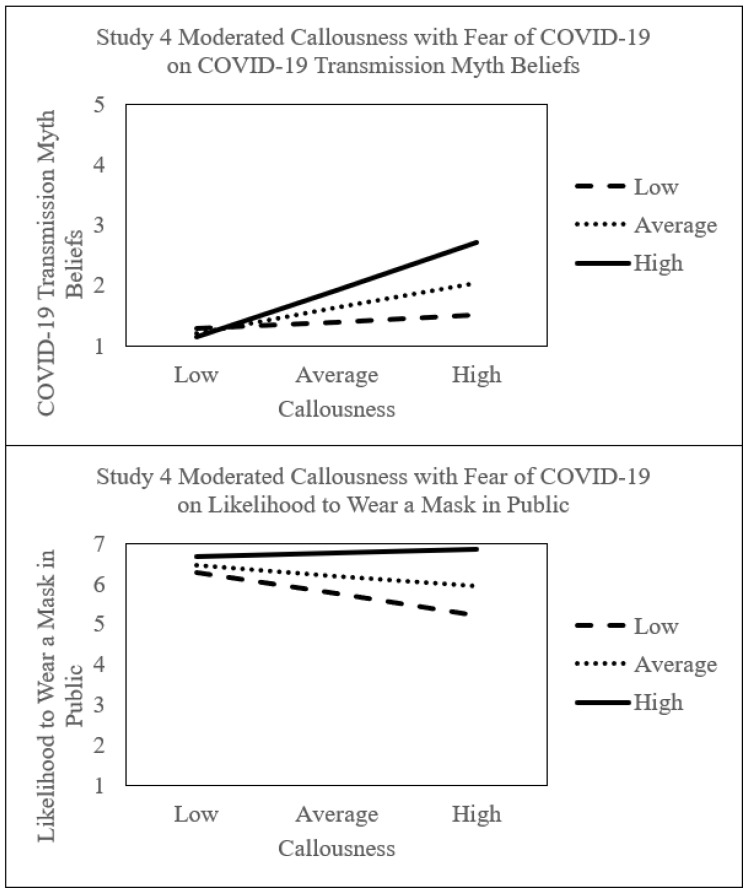
Indirect conditional effects with fear of COVID-19 and callousness.

Figure 3 illustrates moderated mediation results with callousness and fear of COVID-19 on CTMS scores and wearing a mask in public. Results were bootstrapped with the SPSS PROCESS V3.5 10,000 resampling procedure at the 95% confidence interval [128]. Table 7 shows the confidence intervals for the moderated effects. Moderated mediation analysis with low callousness and high fear of COVID-19 attenuated wearing a mask in public (*effect* = 0.085, LLCI 0.056 to ULCI 0.114) (H4). However, high callousness and fear of COVID-19 exacerbated COVID-19 transmission misinformation beliefs (*effect* = 0.224, LLCI 0.163 to ULCI 0.285) (H5). This indirect conditional path decreased wearing a mask in public (*effect* = −0.052, LLCI −0.077 to ULCI −0.032).

## 4. Discussion

Rigorous design and objective procedures refined the large initial set of items into a succinct list. Across four studies, the researchers designed and generated the 12-item CTMS. The CTMS demonstrated high reliability with each dataset. Compared to a wide range of measures, the CTMS evinced uniqueness. The CMTS demonstrated convergent validity with the fear of COVID-19, callousness, generic conspiracy beliefs, risk-taking, and conservatism. Meanwhile, the CTMS diverged with personal vulnerability to disease, compassion, and conscientiousness.

Furthermore, correlational results provided insights into demographic differences. Males, younger aged, college educated, and religiosity associated with greater COVID-19 transmission misinformation beliefs. These results corroborate prior research that found males and younger individuals were less concerned with COVID-19 risks and partaking in health-protective behaviors [132]. For persons without a college degree, understanding of COVID-19 transmission may relate to essential work positions and personal experiences. For example, grocery store workers interacted with enumerate customers and company policies mandated facial covering. Daily, their personal well-being was at risk, and many had to enforce company policies. Many essential workers did not have the luxury to work from home. Community cooperation to public health policies acted as their main source of protection. COVID-19 was perceived as a real health threat to essential workers. First-hand experience and/or knowing people with the disease can change minds.

In addition, viewing more news on average each week correlated with higher CTMS scores. More news does not mean more well informed. In fact, results indicated more misinformation in someone’s cognition. Sorting and decerning fact versus rumor leaned towards believing COVID-19 transmission misinformation. The existence of misinformation in society is an ongoing problem in the battle against COVID-19.

Meanwhile, the strong correlational relationship between callousness and COVID-19 transmission misinformation beliefs may be explained by the lack of willingness to hear and learn from others such as scientists stating to wear a mask to protect others. The researchers mean centered variables in the moderated mediation PROCESS analysis and assessed standardized effects to account for any potential multicollinearity issues [126].

Fear of COVID-19 moderated by callousness increased mask wearing in public. This suggests that early public health messaging can stick with callous individuals. For instance, wearing a mask properly in public acts as a primary physical defense to repel respiratory droplets that carry the COVID-19 virus between people. Such messaging helps to inform the public about the dangers and to take preventative action.

However, mediation analysis illustrated that greater COVID-19 transmission misinformation beliefs decreased mask wearing in public. Moderated mediation results evinced that greater callousness with greater COVID-19 fears are more likely to believe transmission rumors and thereby exacerbated not wearing a mask in public. The inception of information for callous individuals may pose insurmountable reeducation challenges if misinformation beliefs solidify.

Hearsay stating that face coverings do not work arguably resulted in needless infections and lost lives, many through asymptomatic transmission [20]. The use of this measure to study human behaviors can assist in flattening the curve. Research using the CTMS can also provide insights in combating the next cycle of rumors to the future disease that will grip the world.

## 5. Implications

Scientists study the transmission of infectious diseases so that the public can adopt preventative behaviors. Public health officials inform the public with facts from rigorous controlled studies. They design public policies that mitigate risks which have saved countless lives. Despite the rapid testing and release of accurate COVID-19 transmission information by public health officials, misinformation took hold in society. If COVID-19 is falsely believed to not be transmitted by respiratory air droplets, what are these misinformation believers doing to take preventative measures? Across the four CTMS studies, religiosity was positively correlated to the CTMS, indicating religion as a source of misinformation to COVID-19 transmission. Many misinformation believers received COVID-19 information from their religious organizations and believed prayer protected from infection [133,134]. While the act of praying and community provide other benefits, there is no scientifically studied evidence that prayer prevents COVID-19 infections [133]. In fact, large in-person gatherings with bunching in close proximities are known as superspreader events [58,135,136]. The false belief that in-person religious gatherings protect against transmission inadvertently spreads the disease [137,138]. Understanding this logic can help public health officials to reach these groups. The CTMS provides a tool for researchers to identify this subgroup and discover relationships to other variables.

Is misinformation causing losses to providing immediate healthcare or do the losses extend further? The misleading videos filming local hospitals showing empty waiting rooms and parking lots during the COVID-19 pandemic fostered an environment of mistrust in health institutions [54,55]. Although most patients are instructed to quarantine at home because of the highly contagious nature of COVID-19, the misinformation cast doubt on the number of cases and deaths reported by the CDC and reputable health institutions. If hospital ICU beds and respirator units are outnumbered by patients, one could imagine full hospitals resembling a packed football stadium on gameday. This contradictory information posed a problem for health institutions advising the public how the virus is spread and how preventative measures (e.g., face coverings and physical distancing) can save lives. The false empty hospital narrative suggests there is no real pandemic and not to trust health institutions. In addition to the service time lost to provide patient care, transmission misbeliefs contributed to citizens performing risky behaviors that transferred the disease through communal spread (e.g., large indoor gatherings and weddings) [135,138].

Furthermore, there are damaging long-term costs to this public mistrust in health institutions. Given the mutated variants of COVID-19 [139] and shelter-in-place public fatigue [140], the public may resist new developing scientific information. Public health institutions are effective if the public responds and changes behaviors to prevent the spread of disease. Of importance to public health is mitigating risks and public awareness. A lack of public cooperation undermines efforts. For example, facial coverings are known to work if they seal and cover air pathways (e.g., the nose and mouth). Improperly wearing a mask (e.g., exposing the nose while around others) will not effectively prevent contracting COVID-19. So even if the public begrudgingly participates in preventative measures to follow ordinances, individuals may still become sick because they choose to disregard health reports. This scale captures one aspect of COVID-19 misbeliefs that can help researchers study relationships with outcome variables.

Addressing rumors can accompany a dissemination plan. This can increase public understanding and cooperation in health care decisions. Widespread misinformation reduced participation in preventative behaviors (e.g., wearing a face mask in public) because it was believed that transmission was not through respiratory air droplets (e.g., it was misbelieved that transmission was through 5G network towers) [141]. Steffens et al. (2019) recommended openly communicating with evidence-informed responses to misinformation. Safe-space dialogue can help unravel narratives [142].

## 6. Limitations

This study collected survey responses months into the pandemic. The results demonstrated that COVID-19 transmission misinformation beliefs remained consistent and stable. Public health education discouraging the spread of misinformation appeared to stop the creation of widely recognized new COVID-19 transmission falsehoods. However, as time progresses, belief in these rumors may wane as individuals forget. For example, eyewitness recall of incidents can be influenced based on words used to asked to describe what happened [143]. Human memory is malleable and changes with new information [144]. The currency of the CTMS may shift overtime. Although society at large experienced major changes from the COVID-19 pandemic with lasting impressions, memories will fade. This is like forgetting the aftermath of the terrorist attacks of 9/11, events from the last hurricane, or previous Olympic results. They become a distant memory, both present and possible to influence.

While this study measured mask wearing in public through self-reporting, evaluating proper mask wearing that covers someone’s respiratory orifices (i.e., the nose and mouth) requires physical observation. Public health marketing has missed the initial opportunity to explain the purpose and correct way to cover air passages that reduce the transfer of respiratory droplets that carry the COVID-19 virus between people. In the first outbreak, global mask scarcity necessitated any form of mask be created (e.g., cut from t-shirts and garments). Masks varied in thickness and design. Masks can restrict the amount of oxygen breathed in by someone. Some people find them uncomfortable. Therefore, people wear them exposing their nose and covering their chins. Further observational research with the CTMS can reveal more insight into proper public health behaviors and cooperation.

The transmission of information, beliefs, and actions are not inherently linked [145,146]. The existence of rumors does not mean society will believe them or act on them. For example, politicians have capitalized in times of “chaos” for rumors to adhere and generate action [146]. Meanwhile, DiFonzo et al. (2013) studied how a consensus and reinforcement from others increased belief in rumors. The COVID-19 pandemic created unprecedented changes where many people sought out news and explanations in a time of moral panic [14]. Advancements in social media communication and societal lockdowns created an opportunity for unfounded claims to instantaneously spread during the initial outbreak [43,79]. As the pandemic continued, accurate information and debunked claims reduced new rumors from spreading [45,100]. Results from this study reflect a distinct circumstance where society, media, and national leaders experienced a collective period of uncertainty, followed by public health policies designed to mitigate contagion. Researchers are encouraged to holistically evaluate rumors, events, time, existing norms, and observed relationships when drawing inferences.

## 7. Future Research

Consuming media can act as a coping method for changes in our environment. Many sought information on social media platforms and news media outlets to understand COVID-19 which shared misinformation [6,79]. Greater media consumption coincided with added stressors such as homeschooling and job losses. Household commodities such as cleaning supplies provided comfort to consumers [147]. Future studies can investigate how COVID-19 transmission misinformation beliefs directed consumption. With false narratives (e.g., house flies transmit COVID-19), purchasing cleaning products would theoretically not protect someone from infection. It is hypothesized those still purchasing household goods are not transmission misinformation believers because these goods (e.g., hand sanitizers) relate to scientifically proven ways to prevent contraction of the disease.

Moreover, some misinformation believers become entrenched in their false narratives. For example, they may wrongfully believe COVID-19 is a conspiracy from international governments using 5G networks to take away personal freedoms and scientists are their devious agents. How do these misinformation believers react to facts presented on websites such as from the CDC or WHO? MRI studies could reveal which parts of the brain are activated among misinformation believers versus fact believers. It can provide neurological evidence for how misinformation believers react to counternarrative facts. It is postulated that misinformation believers will respond with an emotional rejection of the information visible in brain imaging.

Furthermore, this research can provide insights into how to change incorrect ideas about COVID-19 transmission. Are the CDC and WHO fact pages enough to counter misinformation? If misinformation believers actively reject and seek fake news sources that confirm their false narratives, how can health providers undo this damage? How can health providers practice medicine if misinformation believers are uncooperative? Best practices to dispel misinformation and reeducate are first steps to providing health care. Qualitative research with nurses and doctors who have worked with misinformation believers could catalog effective dispelling techniques. Researchers recommend listening and directly addressing vaccine misbeliefs as an approach [142]. However, are short medical appointments listening and providing facts enough when many actively read unscientific opinion pieces as truths?

While rumors generally have a negative connotation associated with the COVID-19 pandemic, rumors have historically had many benefits for society. Rumors can encourage healthy debate in democratic groups [61]. Rumors of COVID-19 transmission gave scientists reasons to investigate and eventually disprove claims that could have been true (e.g., cold weather will kill off the virus) [28]. Through process of elimination, a large body of evidence-based literature exists clearly debunking how the disease spreads. The list of CTMS items provides a concise list of likely rumors researchers can promptly investigate during the next pandemic. Reaching those callous and entrenched in misinformation communities will impact public health.

Public health officials have spent considerable time creating education materials to counter misinformation in each new health crisis [57,79]. To what degree does education make a difference? How do we educate those more prone to accept misinformation? Unfortunately, transmission inaccuracies are ubiquitous and continue to influence behaviors. For example, some people without preexisting conditions believed they had a degree of invulnerability to COVID-19, although the virus did not discriminate based on genes and current health conditions [13,148]. What kind of education is most effective in undoing rumors? Would teaching general virus reproduction and cellular biology help demystify the infection process, or would directly debunking rumors better serve the public? The laundry list of unfounded claims that wildly spread with new diseases can be circumvented by teaching basic epidemiology [57]. However, some falsehoods appear believable even with a basic understanding of biology. How can health experts spend less time with each new disease reeducating the public and more time saving lives? Future research can utilize the CTMS to identify misinformation believers and which method is more effective in dispelling rumors.

## 8. Conclusions

The CTMS provides a contextual tool in the infodemic battle against COVID-19. The CTMS quantifies the degree and amount of COVID-19 transmission misinformation respondents believe. Greater COVID-19 fear is associated with greater COVID-19 transmission misinformation beliefs, which is associated with lower mask wearing in public. This important tool provides a method for researchers to study behaviors and relationships with other variables. It is valuable to address contemporary problems (e.g., resistance to wearing a mask in public). Variants of COVID-19 exist. Diseases are becoming more deadly (e.g., Ebola) and contagious. The scientific community can expect the next infodemic while also preparing for the next pandemic.

## Figures and Tables

**Table 1:** Sampling adequacy statistics.

	Study 1		Study 2		Study 3		Study 4	
Kaiser–Meyer–Olkin Measure of Sampling Adequacy	0.974		0.970		0.971		0.973	
Bartlett’s Test of Sphericity	χ^2^(66) = 6889.146	***	χ^2^(66) = 7019.133	***	χ^2^(66) = 6270.147	***	χ^2^(66) = 7460.664	***

Note: *** *p* < 0.001.

**Table 2:** Item-factor loadings and item-level descriptive statistics for COVID-19 transmission misinformation scale (CTMS).

		Study 1 (*N* = 597)			Study 2 (*N* = 651)			Study 3 (*N* = 583)			Study 4 (*N* = 602)		
		Alpha = 0.964			Alpha = 0.959			Alpha = 0.960			Alpha = 0.965		
Item		M	(SD)	Factor Loading	M	(SD)	Factor Loading	M	(SD)	Factor Loading	M	(SD)	Factor Loading
(1)	5G mobile networks spread COVID-19	1.70	(1.20)	0.850	1.61	(1.06)	0.815	1.73	(1.19)	0.831	1.61	(1.14)	0.881
(2)	COVID-19 will go away with cold weather	1.79	(1.20)	0.871	1.74	(1.13)	0.878	1.85	(1.24)	0.862	1.65	(1.13)	0.893
(3)	As a foreign disease, only foreigners can catch COVID-19	1.63	(1.20)	0.895	1.53	(1.08)	0.894	1.71	(1.21)	0.899	1.49	(1.11)	0.926
(4)	Drinking bleach will prevent COVID-19 infection	1.61	(1.19)	0.875	1.57	(1.12)	0.888	1.71	(1.18)	0.862	1.49	(1.09)	0.893
(5)	Consuming garlic will prevent COVID-19 contraction	1.95	(1.28)	0.867	1.90	(1.20)	0.840	2.02	(1.27)	0.832	1.81	(1.20)	0.865
(6)	Adding pepper to meals will prevent COVID-19 infection	1.78	(1.21)	0.892	1.75	(1.15)	0.869	1.86	(1.25)	0.862	1.64	(1.14)	0.881
(7)	Hydroxychloroquine is a sure defense from COVID-19 contraction	2.25	(1.31)	0.720	2.29	(1.25)	0.701	2.30	(1.29)	0.705	2.12	(1.23)	0.700
(8)	Drinking hard liquor protects you from COVID-19 infection	1.79	(1.24)	0.877	1.71	(1.15)	0.874	1.81	(1.22)	0.880	1.61	(1.12)	0.875
(9)	Hand dryers kill the COVID-19 virus	2.03	(1.21)	0.790	2.04	(1.17)	0.756	2.06	(1.21)	0.775	1.94	(1.14)	0.774
(10)	If you do not believe COVID-19 exists you will not contract it	1.66	(1.17)	0.877	1.62	(1.17)	0.886	1.80	(1.22)	0.877	1.57	(1.16)	0.921
(11)	If you can hold your breath for a prolonged period, you are COVID-19 virus-free	1.77	(1.23)	0.875	1.73	(1.15)	0.832	1.88	(1.20)	0.846	1.65	(1.10)	0.854
(12)	Houseflies spread COVID-19	2.06	(1.20)	0.772	2.06	(1.15)	0.708	2.10	(1.26)	0.777	1.99	(1.13)	0.728

**Table 3:** Descriptive statistics of items considered COVID-19 transmission misinformation.

	Frequency Believe Not True	Percent Believe Not True	Mean	SD	Skewness	Kurtosis
Item 1	2155	88.57%	1.66	(1.15)	1.59	1.30
Item 2	2114	86.89%	1.76	(1.17)	1.31	0.44
Item 3	2132	87.63%	1.59	(1.15)	1.76	1.67
Item 4	2139	87.92%	1.59	(1.14)	1.77	1.71
Item 5	2043	83.97%	1.92	(1.24)	1.03	−0.27
Item 6	2113	86.85%	1.76	(1.19)	1.36	0.58
Item 7	1973	81.09%	2.24	(1.27)	0.53	−0.93
Item 8	2119	87.09%	1.73	(1.18)	1.44	0.82
Item 9	2082	85.57%	2.02	(1.18)	0.86	−0.40
Item 10	2124	87.30%	1.66	(1.18)	1.66	1.40
Item 11	2122	87.22%	1.76	(1.17)	1.34	0.46
Item 12	2105	86.52%	2.05	(1.18)	0.78	−0.47

Notes: Responses 1—definitely false, 2—probably false, and 3—not sure/cannot decide counted as believe as not true. Responses 4—probably true and 5—definitely true counted as believe as true. Percent out of 2433 COVID-19 transmission misinformation item responses. All misinformation statements had responses that ranged from 1 to 5. Means, standard deviations, skewness, and kurtosis based on the 5-point scale. Each statement was unproven at the time of the study.

**Table 4:** Demographic Characteristics of Participants (*N* = 2433).

Demographic Characteristics	Frequency	Percentage
**Gender**		
Male	839	34.48
Female	1594	65.52
**Age range (years)**		
18–29	467	19.19
30–39	762	31.34
40–49	519	21.33
50–59	399	16.4
60 and over	286	11.76
**Household Income**		
Less than $10,000	111	4.56
$10,000–19,999	172	7.07
$20,000–29,999	240	9.86
$30,000–39,999	325	13.36
$40,000–49,999	276	11.34
$50,000–59,999	349	14.34
$60,000–69,999	186	7.64
$70,000–79,999	177	7.27
$80,000–89,999	152	6.25
$90,000–99,999	131	5.38
$100,000 and over	314	12.91
**Ethnicity**		
Caucasian	1915	78.71
Hispanic/Latino	118	4.85
African American	208	8.55
Native American	26	1.07
Asian	126	5.18
Other	40	1.64

**Table 5:** Bivariate correlations of the 12-item COVID-19 Transmission Misinformation Scale (CTMS) with variables and demographics.

	Study 1				Study 2					Study 3				Study 4		
Variables	M	(SD)	r		M	(SD)	r		M	(SD)	r		M	(SD)	r	
Fear of COVID-19	3.52	−1.72	0.506	***	3.37	−1.64	0.487	***	3.56	−1.61	0.565	***	3.3	−1.61	0.486	***
Callousness	–	–	–		–	–	–		3.03	−1.26	0.789	***	2.35	−1.43	0.736	***
Conscientious	5.06	−1.05	−0.408	***	5	−0.96	−0.422	***	–	–	–	–	–	–	–	
Conservatism	4.43	−0.95	0.164	***	4.37	−0.92	0.139	***	–	–	–	–	–	–	–	
Generic Conspiracy Belief Scale	2.82	−1.01	0.605	***	2.88	−0.99	0.556	***	–	–	–	–	–	–	–	
PANAS	–	–	–		1.25	−1.38	−0.168	***	–	–	–	–	–	–	–	
Perceived Vulnerability to Disease	–								4.31	−0.8	−0.092	***	4.33	−0.84	−0.106	***
Variety Seeking	–								4.51	−1.08	−0.186	***	–	–	–	
Risk Taking	–								2.73	−1.54	0.75	***	2.37	−1.45	0.678	***
Compassion	–												5.46	−1.1	−0.55	***
Wearing a Mask in Public	–												6.36	−1.19	−0.301	***
Demographic Characteristics																
Gender (Female)	1.66	−0.48	−0.265	***	1.66	−0.47	−0.172	***	1.64	−0.48	−0.284	***	1.66	−0.48	−0.279	***
Age	42.34	−13.45	−0.158	***	41.25	−12.93	−0.123	***	41.97	−13.34	−0.183	***	41.06	−13.28	−0.077	***
College Degree	0.71	−0.45	0.218	***	0.69	−0.46	0.147	***	0.72	−0.45	0.258	***	0.69	−0.46	0.121	**
Average Weekly News (Hours)	4.13	−2.65	0.166	***	3.68	−2.64	0.242	***	3.99	−2.62	0.233	***	3.62	−2.66	0.184	***
Religiosity	4.58	−1.93	0.339	***	4.25	−1.93	0.339	***	4.63	−1.85	0.295	***	4.22	−1.99	0.317	***

Note: ** *p* < 0.01, *** *p* < 0.001. Gender was dummy coded with males as 1 and females as 2. College degree was dummy coded with those with an associate degree or higher as 1 and those without 0. Religiosity was measured averaging two items (“Religion/spirituality was an important part of my up bringing” and “I currently consider myself to be a member of a religious or spiritual organization”) on a 7-point scale (strongly disagree—1 to strongly agree—7).

**Table 6:** Study 4 moderated mediation results of fear of COVID-19 and wearing a mask in public.

	Outcome							
	COVID-19 Transmission Misinformation Beliefs				Wearing a Mask in Public			
Antecedent	Coeff.	*SE*	*t*	*p*	Coeff.	*SE*	*t*	*p*
Fear of COVID-19	0.128	0.014	8.973	<0.0001	0.265	0.031	8.456	<0.0001
COVID-19 Transmission Misinformation Scale (CTMS)	—	—	—	—	−0.617	0.084	−7.315	<0.0001
Callousness	0.295	0.018	16.223	<0.0001	−0.156	0.045	−3.486	<0.001
Fear of COVID−19 x Callousness	0.124	0.009	14.202	<0.0001	0.118	0.021	5.678	<0.0001
Covariates								
Gender (female)	−0.16	0.047	−3.407	<0.001	0.139	0.097	1.429	0.154
Age	−0.001	0.002	−0.46	0.645	0	0.003	−0.096	0.924
College Degree	−0.044	0.046	−0.954	0.34	0.07	0.094	0.747	0.456
Weekly Hours of News (Average)	−0.008	0.009	−0.964	0.336	0.023	0.018	1.321	0.187
Religiosity	0.057	0.011	5.11	<0.0001	−0.051	0.023	−2.21	<0.05
Model Summary		R^2^ = 0.727				R^2^ = 0.246		
		F(8, 593) = 197.227, *p* < 0.0001				F(9, 592) = 21.502, *p* < 0.0001		

Notes: The 12-Item COVID-19 Transmission Misinformation Scale was the mediator in the model. Variables were mean centered. Gender was dummy coded with males as 1 and females as 2. College degree was dummy coded 0 without and 1 with an associate degree or higher.

**Table 7:** Study 4 conditional direct and indirect effects with callousness as moderator.

	Left-Leaning	Average	Right-Leaning
	Low (−1 SD)	Mean	High (+1 SD)
Fear of COVID-19 → Wearing a Mask in Public	−0.040 (LLCI − 0.077 ULCI − 0.002)	0.085 (LLCI 0.056 ULCI 0.114)	.333 (LLCI 0.295 ULCI 0.372)
Fear of COVID-19 → CTMS	0.106 (LLCI 0.029 ULCI 0.183)	0.224 (LLCI 0.163 ULCI 0.285)	.460 (LLCI 0.363 ULCI 0.557)
Fear of COVID-19 → CTMS → Wearing a Mask in Public	0.024 (LLCI 0.003 ULCI 0.051)	−0.052 (LLCI − 0.077 ULCI − 0.032)	−0.206 (LLCI − 0.287 ULCI − 0.143)

Note: Bootstrap 10,000 resampled confidence intervals, CTMS = 12-item COVID-19 Transmission Misinformation Scale.

## Data Availability

Due to the nature of this research, participants of this study did not agree for their data to be shared publicly, so supporting data is not available.
